# Hemodynamic Effects of the Light Stabilizer Tinuvin 770 in Dogs **In Vivo**

**DOI:** 10.2174/1874104501812010088

**Published:** 2018-08-31

**Authors:** Miklos Krepuska, Marta Hubay, Endre Zima, Aniko Kovacs, Violetta Kekesi, Huba Kalasz, Brigitta Szilagyi, Bela Merkely, Peter Sotonyi

**Affiliations:** 1 Heart and Vascular Center, Department of Vascular Surgery, Semmelweis University, Budapest, Hungary; 2 Department of Forensic Pathology, Semmelweis University, Budapest, Hungary; 3 Heart and Vascular Center, Department of Cardiology, Semmelweis University, Budapest, Hungary; 4 Hungarian Institute for Forensic Sciences, Budapest, Hungary; 5 Department of Pharmacology and Pharmacotherapy, Semmelweis University, Budapest, Hungary; 6 Department of Mathematical Geometry, Institute of Mathematics, University of Technology and Economics, Budapest, Hungary

**Keywords:** Tinuvin 770, L-type Ca^2+^ channel, Nicotinic acetylcholine receptor, Cardiotoxicity, Circulation, Hemodynamic parameters, dp/dt

## Abstract

**Introduction:**

Tinuvin 770 [bis(2,2,6,6-tetramethyl-4-piperidinyl) sebacate, Ciba-Geigy, Basel, Switzerland] is a UV light stabilizer that is a component of many plastic materials used world-wide in the medical and food industries. We report on the acute hemodynamic effects of Tinuvin 770 examined in dogs.

**Materials and Methods:**

Tinuvin 770 was dissolved in a mixture of saline and ethanol (1:1 v/v) and was administered to 12 intravenously narcotized and respirated dogs in increasing doses (T1-T7: 1, 3.3, 6.6, 10, 33.3, 66.6 and 100 mg, respectively). The doses were given as bolus injections over a three minute period, and the effects were recorded for 12 minutes. The vehicle was used as a control. Hemodynamic parameters (heart rate, blood pressure, end-diastolic pressure, dp/dt, cardiac output) and ECG were monitored continously.

**Results:**

At doses T1-T4, systolic and diastolic blood pressures, mean pressure and ventricular contractility were significantly decreased without significant changes in cardiac output, heart rate, or PQ interval. At doses T5 and T6, declines in blood pressure and myocardial contractility were observed. At doses T6 and T7, heart rate and PQ interval decreased substantially. Irreversible circulatory failure occured in one dog after administering dose T6 and in 8 dogs following dose T7.

**Conclusion:**

Tinuvin 770 induces acute hemodynamic alterations. In lower doses, it causes peripheral vasodilatation, however at higher doses acute cardiac failure occured. Plastics containing Tinuvin 770 should be used with care in medical practice and the laboratory.

## 1 INTRODUCTION

Tinuvin 770 is widely used to stabilise polyethylene [1], polypropylene, polycarbonate, polyurethane, polystyrene, polyamide, polyacetyl and acrylonitril polymer products [2]. It belongs to the group of sterically Hindered Amine Light Stabilizers (HALS) [3, 4]. It is preferencially used in stabilizing thin plastic films and membranes used in medicine and food-industry [5]. Glossman and his colleagues reported that Tinuvin 770 [bis (2,2,6,6-tetramethyl-4-piperidinyl) sebacate, Ciba-Geigy, Basel, Switzerland], a UV stabilizer [6], can be eluted from plastic laboratory tubes. They showed that the compound is a potent (IC_50_ values < 10nM) L-type Ca^2+^ channel blocker [7]. Tinuvin 770 is structurally different from other Ca^2+^ channel blockers [8, 9] it acts by binding to the phenylalkylamine and benzothiazepine selective domain of the α1 subunit of the L-type Ca^2+^ channel, and was shown to decrease the 1,4-dihydropiridine-sensitive Ca^2+^- uptake [7]. The tissue specificity of previously known Ca^2+^ antagonists is quite different. Dihydropiridines (like Nifedipine) act primarily on the smooth muscle cells of peripheral vessels, inducing dilatation of the arterioles and coronaries and producing decreased afterload [9]. Dihydropiridines do not affect atrial-ventricular conduction and slightly decrease myocardial contractility can only be observed following high doses. Their intense peripheral vasodilatory effect may result in reflex tachycardia [10, 11]. Phenylalkylamines (*e.g*. Verapamil) act mainly on the myocardium. Benzothiazepines produce only weak vasodilatory effects and decrease contractility compared to dihyropiridine. The action of was shown to be similar to that of of benzothiazepines *in vitro*.

Papke *et al*. [12] showed that Tinuvin 770, dissolved from plastics used in medical practice, blocks α4, β2 subunit-containing nicotinic acetylcholine receptors in the central nervous system more potently (IC_50_ ≈200 nM), than the nicotinic ganglion blocker mecamylamine (IC_50_ ≈10 μM) [13-15]. Tinuvin 770 failed to decrease blood pressure and nociceptive threshold either, and did not alter locomotor activity [15].

We have previously demonstrated that *in vitro* Tinuvin-770 has time-dependent cardiotoxic effects on isolated myocardial cells [16] and dose-dependent chronic cardiotoxicity in rats *in vivo* [17]. It was also shown that Tinuvin 770 increased intracellular Ca^2+^ accumulation and catecholamine release similarly to the first generation L-type Ca^2+^ blockers.

In the present study, we aimed to analyze the acute haemodynamic and cardiac effects of Tinuvin 770 in a dog model. The experimental animal model used is appropriate to study the hemodynamic effects, to perform electrophysiological monitoring and to determine the dose-effect relationships.

## 2 MATERIALS AND METHODS

### 2.1 General Interventions

Acute hemodynamic experiments were performed on 12 mongrel dogs (weight: 24±1 kg, 7 males, 5 female). The investigation conformed to the Guide for the Care and Use of Laboratory Animals published by the US National Institutes of Health (NIH Publication No. 85-23, revised 1985) and was in accordance with the ethical regulations of animal care at Semmelweis University. The Regional Ethical Committee of the Semmelweis University approved this experimental study under No: 42/1999/.

### 2.2 Particular Intervention

Each animal was initially anaesthetized with intravenous pentobarbital sodium (Nembutal, CEVA, and 30 mg/kg) and additional anesthesia was given as needed to maintain an appropriate constant level of narcosis. After tracheal intubations, the dogs were respirated with room air by a Cape CV2424 respirator (Cape Engineering Co. Ltd.). The right femoral vein was used to administer Tinuvin 770. One of the femoral arteries served for continuous arterial blood pressure monitoring (Experimetria HG 01 modem, Statham P23Db). The right carotid artery was prepared and a pigtail catheter was introduced for left ventricular pressure and contractility (dp/dt) measurements (Pigtail Catheter 4F, Cordis and Experimetria HG 01 modem, Statham P23Db). The left internal jugular vein was used to measure cardiac output using a wedge pressure balloon catheter, through which 10 ml 10 – 16 °C saline solution was injected at the time of each measurement. A thermistor catheter was introduced into the aorta through the left femoral artery to measure Cardiac Output (CO). Cardiac output was then calculated on the basis of thermodilution. Measurements and calculations were performed with Experimetria CO-100, Cardiostar equipment. Standard surface ECG recording (Madaus Schwarzer CU12) was used to calculate heart rate and measure PQ-intervals.

A preliminary experiment was carried out to determine the effective dose range of Tinuvin 770. A wide range of doses (a doses covered six magnitude of concentrations between the lowest and highest dose: 1 μg – 100 mg) was applied. This preliminary experiment was performed on two dogs. These animals were injected by six doses of Tinuvin 770 (1 μg, 10 μg, 100 μg, 1 mg, 10 mg, 100 mg) and by vehicle as control (described in the next section). Tinuvin 770 in doses lower than 1 mg had neither hemodynamic nor electrophysiologic effects; however, none of the dogs survived the 100 mg dose. Therefore, in the present study the following doses of Tinuvin 770 were used; T1:1mg, T2:3.3mg, T3:6.6mg, T4:10mg, T5:33.3mg, T6:66.6mg, T7:100 mg.

### 2.3 Experimental Protocol

After surgical instrumentation, each animal underwent a 20-minute equilibration period. During the next 20-minute baseline period, haemodynamic parameters and ECG were continuously recorded. Administration of Tinuvin 770 was started after baseline stabilization was achieved. First, vehicle (a mixture of equal amount of saline solution and ethanol) was given intravenously. Then doses T1 – T7 (1; 3,3; 6,6; 10; 33,3; 66,6 és 100 mg of Tinuvin 770 in 5 ml vehicle) were injected over a 3-minute period. Hemodynamic measurements and ECG monitoring were performed at 0, 3, 6, 9, 12, and 15 minutes following Tinuvin 770 injections. Each dog received all the 7 doses of Tinuvin 770 in increasing order.

### 2.4 Statistical Analysis

Data are expressed as means ± SDs. For statistical comparisons repeated measures ANOVA was used. Sphericity was evaluated using Mauchly’s test. If sphericity was not rejected, a Dunnett test was used for post hoc comparisons of the first to the other time points. If sphericity could not be supposed, simple contrasts with Bonferroni correction were evaluated. Values of P<0.05 were considered significant.

## 3 RESULTS

Following the administration of Tinuvin 770 in doses of T1 – T6, there were statistically significant changes in every parameter measured. One dog died at 12 minutes after the T6 dose was given because of irreversible circulatory failure. At dose T7, lethality was 82%: Three dogs died within 3 minutes, and another six died within 6 minutes. Only two animals survived this dose.

### 3.1 Systolic Arterial Pressure

**Fig. (1):**
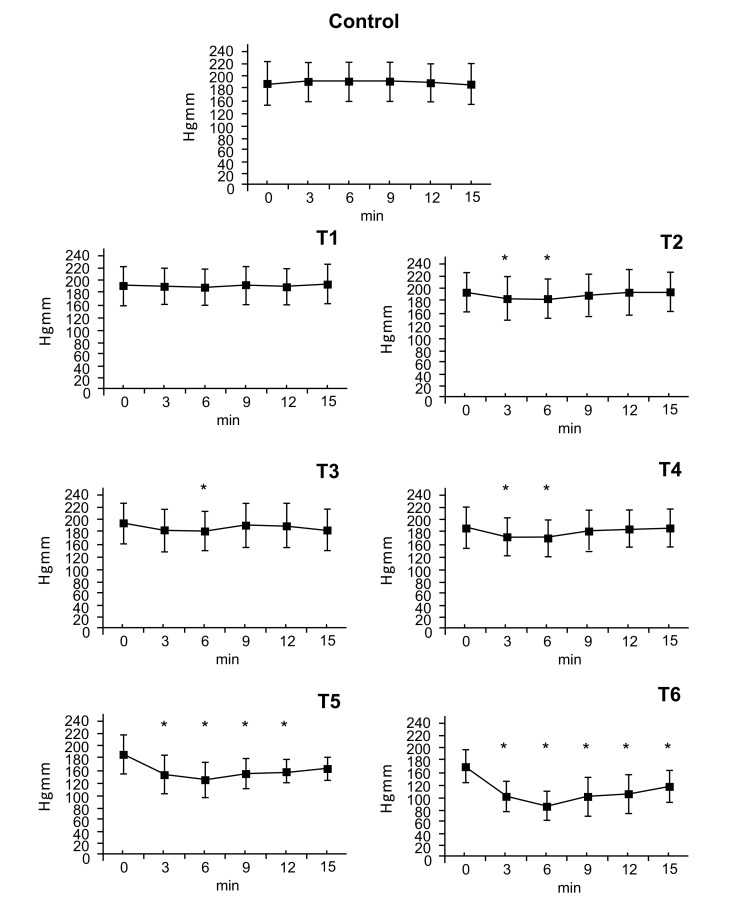
Effects of *Tinuvin 770* on systemic systolic blood pressure (Hgmm). Statistical significance is marked with * (P<0.05).

There were no statistically significant blood pressure changes at the T1. At the T2 dose a small effect on blood pressure was measured at 3 minutes (T2: ∆%≈ -0,058) that ended at 6 minutes. After doses of T3 and T4, an effect on blood presure was seen at 3 minutes with maximum at 6 minutes (T3: ∆%≈ -0.064, T4: ∆%≈ -0.108) that disappeared by 9 minutes. After doses of T5 and T6 blood pressure decreased at 3 minutes, reached a nadir at 6 minutes (T5: ∆%≈ -0,302, T6: ∆%≈ -0,460) and remained low afterwards. By 15 minutes, systolic pressure had nearly returned to normal value (T5: ∆%≈ -0,143 T6: ∆%≈ -0,265) (Fig. **1**).

### 3.2 Diastolic Arterial Pressure

**Fig. (2):**
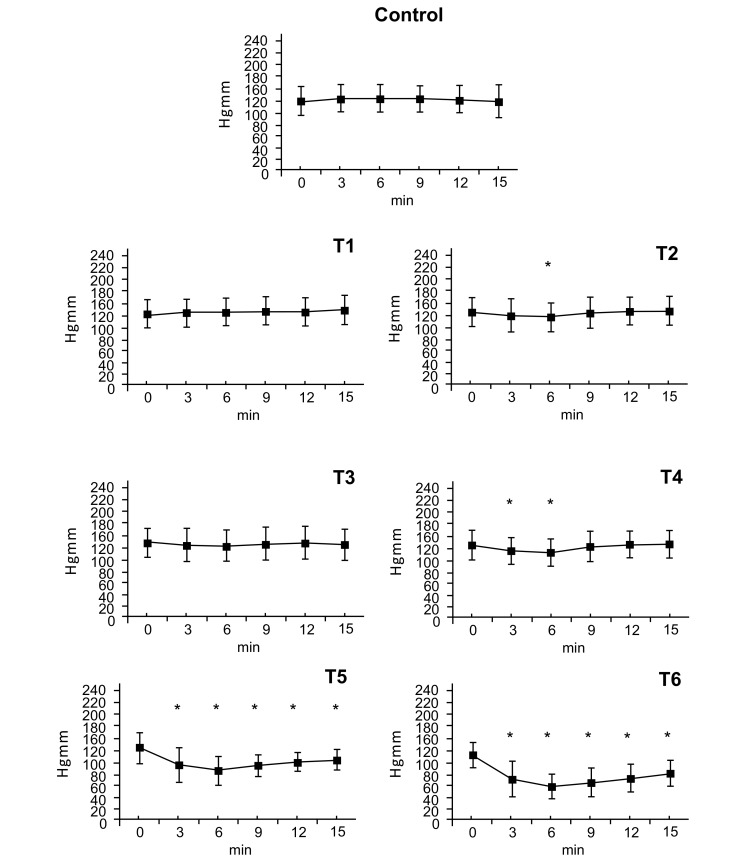
Effects of *Tinuvin 770* on systemic diastolic blood pressure (Hgmm). Statistical significance is marked with * (P<0.05).

There were no statistically significant changes in diastolic blood pressure after the T1dose. Following doses of T2 and T3 there were small decreases in diastolic pressure at the 6-minute timepoint (T2: ∆%≈ -0.067, T3: ∆%≈ -0.052). The T4 dose caused a decrease in diastolic pressure at 3 and 6 minutes (T4: ∆%≈ -0.118). Doses of T5 and T6 caused decreases in diastolic pressure that began at 3 minutes, peaked at 6 minutes (T5: ∆%≈ -0.319, T6: ∆%≈ -0.463) and gradually wore off afterwards (T5: ∆%≈ -0.163, T6: ∆%≈ -0.272) (Fig. **2**).

### 3.3 Mean Blood Pressure

Neither the vehicle nor the T1 dose had an effect on mean blood pressure. The T2 dose decreased mean blood pressure after 6 minutes (T2: ∆%≈ -0.0601); the effect disappeared within 3 minutes. Doses of T3 and T4 (T3: ∆%≈ -0.071, T4: ∆%≈ -0.111) caused a blood pressure decrease within 3 minutes, that further decreased by 6 minutes however the effect disappeared by 9 minutes. Doses of T5 and T6 decreased mean blood pressure within 3 minutes. This effect peaked at 6 minutes (T5: ∆%≈ -0.298, T6: ∆%≈ -0.467) and persisted until the end of the 15 minutes observation period.

### 3.4 Positive dp/dt Values

**Fig. (3):**
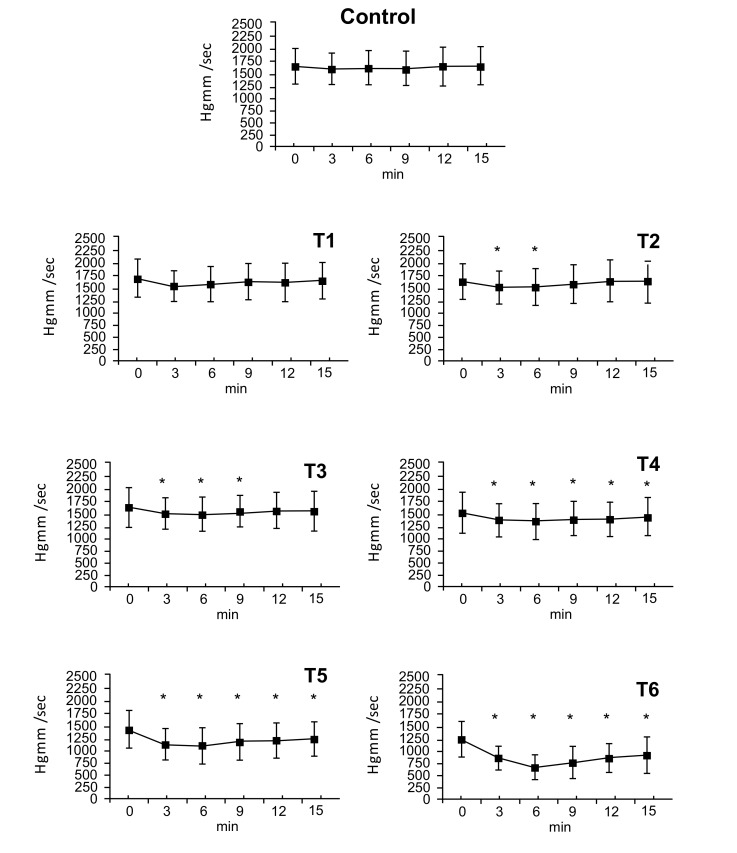
Effects of *Tinuvin 770* on positive dp/dt (Hgmm/sec). Statistical significance is marked with * (P<0.05).

Administration of the vehicle and theT1 dose had no effect on positive dp/dt values. The T2 dose had a maximum effect at 3 minutes (T2: ∆%≈ -0.094), which persisted by the 6 minute timepoint, at 9 minutes no differences were observed. The T3 dose also had a maximum effect at 3 minutes (T3: ∆%≈ -0.101), that ended within 12 minutes. At doses of T4, T5 and T6 the effects on positive dp/dt began at 3 minutes, reached a maximum at 6 minutes (T4: ∆%≈ -0.123, T5: ∆%≈ -0.238, T6: ∆%≈ -0.486) and lasted until the end of the experiment. By 15 minutes, values had nearly returned to initial values (T4: ∆%≈ -0.066, T5: ∆%≈ -0.155, T6: ∆%≈ -0.263) (Fig. **3**).

### 3.5 Negative dp/dt Values

There were no changes following administration of vehicle and the T1 dose. The T2 dose elicited a decrease in negative dp/dt (∆%≈ -0.082) that disappeared by 9 minutes. The maximal effect of the dose of T3 appeared at 3 minutes (T3: ∆%≈ -0.093) and ended by 9 minutes. At doses T4, T5, and T6 changes were seen from 3 minutes until the end of the experiment (T4: ∆%≈ -0.088, T5: ∆%≈ -0.117, T6: ∆%≈ -0.475). At 15 minutes values had almost returned to the 0 time point levels (T4: ∆%≈ -0.060, T5: ∆%≈ -0.113, T6: ∆%≈ -0.309). Since the negative dp/dt is equal to the positive dp/dt proportion, only the positive values are presented in Fig. (**3**).

### 3.6 Heart Rate

Vehicle and dose T1 had no effect on heart rate. Dose T5 decreased heart rate at timepoints 12 and 15. (T5: ∆%≈ 0.039). Dose T6 decreased the rate by minute 9, and this effect persisted until 15 minutes (T6: ∆%≈ 0.108) (Table **1**).

### 3.7 Atrio-Ventricular Conduction

We characterized atrio-ventricular conduction by measuring the PQ interval. Vehicle and the T1-T3 doses had no effect on the PQ interval. Following T4 dose PQ interval decreased at 12 minutes (T4: ∆%≈ 1.025). The PQ decreasing effect of the Tinuvin 770 T5 and T6 doses began at 3 minutes. At dose T5 the PQ decrease reached a minimal at 12 minutes (T5: ∆%≈ 1.084), and the dose of T6 reached a minimal at 9 minutes. Following the T5 and T6 doses, the change in the PQ interval returned almost to the initial value at 15 minutes (T5: ∆%≈ 1.059, T6: ∆%≈ 1.088) (Table **2**).

### 3.8 End-Diastolic Left Ventricular Pressure

There was no change in the End-Diastolic left ventricular Pressure (EDP) at doses T1 and T2. The mean pressure values were around 7 mmHg. However following T3 and T4doses, the EDP values increased to more than 10 mmHg (T3: ∆%≈ 1.264, T4: ∆%≈ 1.385) and no further increase was observed at doses of T5 and T6. The effect of dose T7 could not be estimated.

### 3.9 Cardiac Output

There were no statistically significant changes in Cardiac Output (CO) up to dose of T6. Animals that died soon after the administration of the T7 dose showed a significant decrease in cardiac output. The 3 minute values (T7: ∆%≈ 0.244) could only be collected from 9 dogs, since three dogs died soon after the T7 dose of Tinuvin 770 was administered. By 6 minutes, six more dogs were lost, so there were not enough survivors for statistical analysis.

### 3.10 Systemic Vascular Resistance (SVR)

**Fig. (4):**
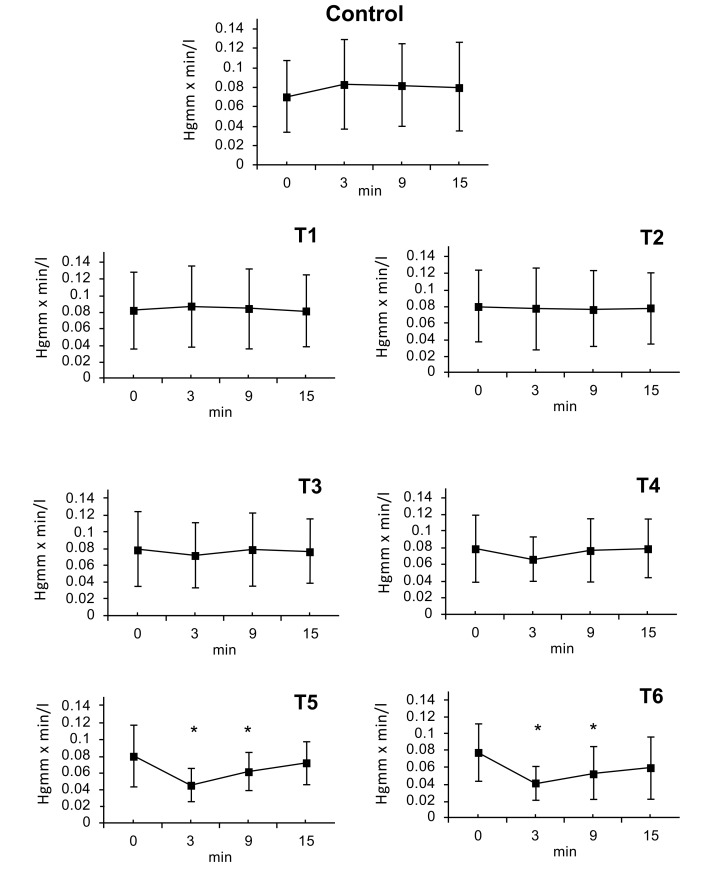
Effects of *Tinuvin 770* on systemic vascular resistance ([HgmmXmin]/l). Statistical significance is marked with * (P<0.05).

SVR was calculated from Mean Arterial Pressure (MAP) and Cardiac Output (CO). SVR=MAP/CO (HgmmXmin/l). There were no statistically significant changes following doses of T1, T2 or T3. At dose T4 a decrease of SVR was observed after 3 minutes (T4: ∆%≈ -0.11); that lasted till 9 minutes. After the doses of T5 and T6, a maximal decrease in SVR was seen at 3 minutes; (T5: ∆%≈ -0.44, T6: ∆%≈ -0.49) this effect slowly regressed by 6 minutes (T5: ∆%≈ -0.23, T6: ∆%≈ -0.33) and the SVR value nearly returned to initial normal value by the 15 minute timepoint (T6: ∆%≈ -0.25) (Fig. **4**).

Circulatory failure was predicted by ischemic changes on the ECG record (negative T wave, ST depression or elevation), by ventricular extra systoles, and also by ventricular fibrillation.

## 4 DISCUSSION

The dose range determined in our preliminary experiment seemed to be appropriate to characterize the acute hemodynamic and cardiac effects of Tinuvin 770. We did not detect statistically significant hemodynamic or electrophysiological effects of the compound at the lowest dose (1mg) studied. In contrast, 75% of the dogs suffered irreversible circulatory failure and died following the administration of the highest Tinuvin 770 dose (100 mg). Hemodynamic effects of Tinuvin 770 were shown dose-dependent. Tinuvin 770 caused changes in hemodynamic and ECG parameters resembles that of typical Ca^2+^ antagonist compounds, although not identical [10, 11]. Dihydropyridines, (for example Nifedipin) due to their vascular selectivity, mainly initiate vasodilatation and their impact on the heart is substantially weaker. Phenylalkylamines (like Verapamil) are considered cardioselective effectively decreasing PQ, decreasing heart rate, and causing a negative chronotropic effect. The actions of the benzothiazepines (like Diltiazem) are also localized mainly on the heart, although their cardioselectivity does not reach that of phenylalkylamines. Chronic cardiotoxicity caused by Tinuvin 770 was also shown in our previous *in vitro* and *in vivo* studies [16, 18]. In our present study, there were dose-dependent decreases both in systolic and diastolic blood pressure values beginning from the dose of T2, and dose T6 caused a 46 percent fall in blood pressure. At dose T7 blood pressure dropped to zero in 9 of the 12 dogs when their heart failed. While changes in myocardial contractility paralleled those in blood pressure at lower doses, an unequivocal drop in contractility was seen at dose T5 (33.3 mg), and this became quite pronounced (-40%) following dose T6 (66.6 mg). Negative and positive dp/dt values were observed. The decrease of positive dp/dt up to the T5 dose exceeded the decrease of the negative dp/dt. This difference was most pronounced at dose T5, where the positive dp/dt denoted a twice as high downfall than the negative one. At dose T6 (66.6 mg) the drop for both parameters were nearly the same. At dose T7 contractility was immeasurable. Higher T3 and T4 doses (6,6; 10 mg) elicited an end diastolic left ventricular pressure increase of 10 Hgmm that did not increase further at higher doses (T5 and T6). A statistically significant acute decrease in heart rate was only seen at doses of T5 and of T6 (33.3 and 66.6 mg). The decline reached 10 percent at the dose T6. A decrease in PQ interval (not exceeding 10%) could also only be detected at doses of T5 and T6 (33.3 and 66.6 mg). Tinuvin 770 at doses of T1-T6 (1-66.6 mg) did not influence cardiac output. However, the T7 dose (100 mg) resulted in circulatory failure associated to a marked fall in CO. Only three of 12 dogs were able to compensate the 100 mg dose of Tinuvin 770 and their circulatory parameters returned to baseline values only after long period (60min) of time. The ECG records showed also signs of severe ischemia. Since cardiac output was found constant, the values of SVR reflect changes in mean arterial pressure at doses of T1-T5. SVR declined in parallel with CO.

Tinuvin 770 dissolved from plastic containers can be absorbed in the human body [19]. It may enter the circulation following oral administration, or can be absorbed through the skin, mucosal membranes, or can directly reach circulation via bandages, catheters, cannulas, *etc*.

Haemodialysis treatment seems to have the danger. Tinuvin 770 contamination of the body can be facilitated by the 1-1.8 square meter surface area of the dialysis membrane, the 250-300 ml/min rate of blood flow, and the repeated treatments [20]. It is worth mentioning, however, that few of the complications associated with hemodialysis treatments have unequivocally been correlated with the dialysis membranes. Reported hemodyalisis associated adverse effects include intoxication caused by chemicals used for sterilization [21, 22], pyrogenic reactions [23], membrane-medication interactions [24], and some specific reactions that developed when certain specific membranes were used [25-27]. Among acute hemodyalisis complications, hypotension, that develops during or immediately after dialysis treatment, has 15-20 percent prevalence [28]. Clinical symptoms (vomiting, dizziness, loss of consciousness) appear together with a drop of blood pressure by 30-50 Hgmm. Concomitant factors are decreased blood volume, reduced peripheral vasoconstriction and cardiac factors (known ischemic heart disease, arrhythmia, diminished left ventricular function) [29, 30].

Tinuvin 770 is present in different dialysis membranes is extracted by aqueous phase plasma-like solutions [18], was shown to act as an L-type Ca2+ channel blocker. It could contribute to the development of dialysis-related hypotension through its cardiovascular and central nervous system effects.

Our present *in vivo* results summarized above support our earlier *in vitro* observations that Tinuvin 770 is an effective L-type Ca^2+^ channel inhibitor. However, its mode of action seems to be complex being closest to that of the benzothiazepines (*e.g*. Diltiazem), *vs* Phenylalkylamines (*e.g*. Verapamil) or dihydropiridines (*e.g*. Nifedipin). Its actions are not identical to those of any clinically used L-type Ca^2+^ channel blocker. At lower doses (3.3 – 10 mg), it primarily has peripheral effects. Mild vasodilatation helps the pumping function of the heart, decreasing after-load accelerates motion of the left ventricle, and it decreases tension of the ventricular wall and improves coronary circulation. These effects are supported by the data presented, namely that the initially increasing EDP is not rising any further. However, at higher doses, its acute cardiac effects become more marked. A gradual decrease in ventricular contractility lead to cardiovascular failure – as observed following the administration of the extra high doses of T6 andT7 (66.6 and 100 mg) of Tinuvin 770. Atrio-ventricular conduction was only influenced significantly at higher doses, but without clinically significant alterations in the heart rate. Besides its negative inotropic and peripheral vasodilatative effects, at high subtoxic doses, an acute negative chronotropic effect can also contribute to its complex effect due to the increased atrio-ventricular conduction time observed. On the basis of the dose range tested, EC50 (EC_50_ ≤ 0137 mg/body weight) and LD50 (LD_50_ ≤ 4 mg/ body weight) were calculated. Based on the data presented, further studies are needed to clarify the exact mode of action of Tinuvin 770 on cardiac function.

## CONCLUSION

Our present results show that the L-type Ca^2+^ channel inhibitory effect of Tinuvin 770 suggested by Glossmann *et al*. [7] could be demonstrated in an *in vivo* experimental setting examining acute hemodynamic parameters in dogs. We also showed that Tinuvin 770 causes acute cardiac failure at higher doses administered. In further studies, we plan to determine those factors that facilitate dissolution of Tinuvin 770 in cardioactive amounts from dialysis membranes and other medical plastics that may produce acute hemodynamic alterations in patients.

## Figures and Tables

**Table 1:** Heart rate in relation to time following administration of different doses of *Tinuvin 770*. T1-T7 symbols represent 1, 3.3, 6.6, 10, 33.3, 66.6, and 100 mg of Tinuvin 770, respectively. Statistically significant differences are marked with bold numbers and * (P<0.05).

**Time (minutes)**	**Vehicle**	**T1 dose**	**T2 dose**	**T3 dose**	**T4 dose**	**T5 dose**	**T6 dose**
**0**	160,6 ±6,5	155,9 ±5,8	162,8 ±4,6	162,8 ±4,9	156,3 ±6,3	153,7 ±5,6	147 ±6,5
**3**	158 ±5,3	155,2 ±5,3	163,2 ±4,4	165,7 ±4,9	157,5 ±6,7	159 ±8,0	148 ±8,8
**6**	158,4 ±5,8	154,1 ±5,4	163,1 ±4,3	165,3 ±5,1	156,5 ±6,3	156,4 ±8,2	142,9 ±8,8
**9**	158,1 ±6,3	156,4 ±5,6	163,7 ±4,8	164,2 ±5,0	154,5 ±6,1	151,4 ±7,5	136 ±7,9*
**12**	157,5 ±6,3	156,9 ±5,7	163,3 ±4,6	163,5 ±5,7	152,9 ±6,2	148 ±6,7*	135 ±8,4*
**15**	156,7 ±6,0	156,5 ±5,4	163,6 ±4,8	162,7 ±6,1	151,2 ±6,3	147 ±6,6*	131 ±9,6*

**Table 2:** Atrio-ventricular conduction. Extension of PQ interval in relation to time following different doses of *Tinuvin 770* in dogs T1-T7 symbols represent 1, 3.3, 6.6, 10, 33.3, 66.6, and 100 mg of Tinuvin 770, respectively. Statistically significant differences are marked with bold numbers and * (P<0.05).

**Time (minutes)**	**Vehicle**	**T1 dose**	**T2 dose**	**T3 dose**	**T4 dose**	**T5 dose**	**T6 dose**
**0**	0,114±,003	0,118±,003	0,114±,003	0,117±,004	0,120±,003	0,118±,004	0,124±,004
**3**	0,113±,003	0,120±,004	0,117±,004	0,119±,005	0,120±,004	0,125±,004*	0,129±,006*
**6**	0,114±,003	0,123±,003*	0,119±,003	0,120±,004	0,122±,004	0,125±,004*	0,135±,006*
**9**	0,114±,003	0,122±,004	0,120±,004	0,119±,004	0,122±,004	0,126±,005*	0,136±,007*
**12**	0,114±,003	0,119±,003	0,119±,004	0,119±,004	0,123±,004*	0,128±,004*	0,135±,007*
**15**	0,114±,003	0,118±,003	0,117±,004	0,117±,004	0,122±,004	0,125±,004*	0,135±,008*
